# Electroplex as a New Concept of Universal Host for Improved Efficiency and Lifetime in Red, Yellow, Green, and Blue Phosphorescent Organic Light‐Emitting Diodes

**DOI:** 10.1002/advs.201700608

**Published:** 2017-12-01

**Authors:** Wook Song, Jun Yeob Lee, Yong Joo Cho, Hyeonghwa Yu, Hany Aziz, Kang Mun Lee

**Affiliations:** ^1^ School of Chemical Engineering Sungkyunkwan University 2066, Seobu‐ro, Jangan‐gu Suwon Gyeonggi 16419 South Korea; ^2^ Department of Electrical and Computer Engineering and Waterloo Institute for Nanotechnology University of Waterloo 200 University Avenue West Waterloo Ontario N2L 3G1 Canada; ^3^ Department of Chemistry Kangwon National University 1, Gangwondaehak‐gil Chuncheon Gangwon 24341 South Korea

**Keywords:** electroplex, lifetimes, mixed hosts, phosphorescent devices

## Abstract

A new concept of host, electroplex host, is developed for high efficiency and long lifetime phosphorescent organic light‐emitting diodes by mixing two host materials generating an electroplex under an electric field. A carbazole‐type host and a triazine‐type host are selected as the host materials to form the electroplex host. The electroplex host is found to induce light emission through an energy transfer process rather than charge trapping, and universally improves the lifetime of red, yellow, green, and blue phosphorescent organic light‐emitting diodes by more than four times. Furthermore, the electroplex host shows much longer lifetime than a common exciplex host. This is the first demonstration of using the electroplex as the host of high efficiency and long lifetime phosphorescent organic light‐emitting diodes.

## 1 Introduction

Long‐term operational stability is one of the most challenging issues in the field of organic light‐emitting diodes (OLEDs) because of the intrinsic chemical instability of organic or organometallic emitters.^1^ Although the operational stability of OLEDs has reached lifetimes that are required for commercial applications, there is still strong demand to increase the lifetime of green and blue phosphorescent OLEDs (PhOLEDs) because their lifetime is not long enough for some practical applications like large size TVs.^2^

There have been many device and material approaches to improve the long‐term operational stability of green PhOLEDs.^3^ In the device approach, triplet exciton and polaron managing device technology was developed because triplet–triplet annihilation (TTA) and triplet–polaron annihilation (TPA) are the major degradation pathways of PhOLEDs.[[qv: 2b,4]] The TTA process is related to triplet exciton distribution in the emitting layer, and a wide emission zone helps suppressing the TTA mechanism. The TPA process is related to triplet excitons and polaron density in the emitting layer, and reducing extra polarons is an effective way of avoiding the TPA degradation mechanism.

Among the many device designs used to achieve high quantum efficiency (QE) and long operational stability, a mixed host device that adopts a hole transport type host and an electron transport type host would be a strong candidate to avoid the TTA and TPA degradation mechanisms.^5^ Two types of mixed hosts, i.e., exciplex free mixed hosts and exciplex mixed hosts, are known to provide high efficiency and long lifetimes in red, green, and blue PhOLEDs.^6^ However, exciplex free hosts were not useful to improve the lifetime of PhOLEDs due to TPA by charge trapping.^7^ Exciplex hosts were effective in green PhOLEDs,^8^ but they have rarely been used in blue PhOLEDs. In fact, only a few exciplex hosts for blue PhOLEDs have been described because it is difficult to develop a high triplet energy exciplex host due to redshift in the emission energy.^9^ Until now, no lifetime results addressing the blue exciplex host have been reported. Moreover, the host materials adopted in the high energy exciplex host had low bond dissociation energy functional groups like sulfone and dipheylphosphine oxide. Therefore, an advanced host system that can extend the lifetime of green and blue PhOLEDs by overcoming the weaknesses of the current mixed hosts is strongly desired.

In this work, we developed a new host system, dubbed an electroplex host, as an approach to universally improve the lifetime of red, yellow, green, and blue PhOLEDs. A common hole transport material, 4,4′‐di(9H‐carbazol‐9‐yl)‐1,1′‐biphenyl (CBP), and an electron transport material, 2,8‐bis(4,6‐diphenyl‐1,3,5‐triazin‐2‐yl)dibenzo[b,d]furan (DBFTrz), were mixed to form the electroplex host for red, yellow, and green PhOLEDs. In addition, 3,3‐di(9*H*‐carbazol‐9‐yl)biphenyl (mCBP) and DBFTrz were mixed to form the electroplex host for blue PhOLEDs. We demonstrated that the electroplex host performed much better than common exciplex free mixed host and exciplex host systems, and provided improved lifetimes in red, yellow, green, and blue PhOLEDs. Lifetime improvements in the electroplex host device of more than four times were observed compared to common mixed host and exciplex host devices. A detailed emission mechanism study revealed that energy transfer from the electroplex host to the triplet emitter is the dominant emission process. This is the first work demonstrating the use of an electroplex as a host in PhOLEDs.

## 2 Results and Discussion

Electroplexes have been reported in several publications, but they were not used in OLEDs because of the poor emission efficiency of the electroplex and difficulty managing the electroplex formation in the devices.^10^ However, the electroplex can be emitted from an intermolecular complex of two materials and transfer the energy to other materials under an electric field like an exciplex.^11^ An electroplex is a relatively weak intermolecular complex with a weak binding energy compared to an exciplex, which can make it easy to develop a high emission energy blue electroplex. In an exciplex, the high exciton binding energy due to the strong binding of two molecules leads to a large decrease in the exciplex emission energy relative to the emission energy of each host participating in the exciplex formation. In contrast, the exciton binding energy of the electroplex is relatively small compared to that of the exciplex, which facilitates the development of a high emission energy electroplex. Therefore, electroplexes can be used as a host in green and blue PhOLEDs, which require high emission energy in the host material. In addition, the underlying concept of using the electroplex as the host of PhOLEDs is to take advantage of an energy transfer process for improved lifetimes in the emission process of dopant materials like exciplexes. The electroplex can behave like an exciplex when used as a host material. Furthermore, the electroplex can provide high emission energies for green and blue PhOLEDs.

Two host materials should be combined to develop the electroplex host. As the electroplex is an intermolecular complex formed under an electric field, the two hosts should have opposite electron donating and accepting characteristics. Therefore, an electron donor‐type hole transport host and an electron acceptor‐type electron transport host are required to prepare the electroplex host. Moreover, the electron donating and accepting character of the hosts, which is reflected in the highest occupied molecular orbital (HOMO) and the lowest unoccupied molecular orbital (LUMO), respectively, must be properly managed. Excessively small or large differences in the HOMO and LUMO offset between the two hosts can lead to an electroplex free host. Therefore, a carbazole‐type host and a triazine‐type host were chosen as the host materials to construct the electroplex host. Carbazole‐type hosts like CBP and mCBP have HOMO/LUMO around −5.9 to −6.1/−2.4 to −2.6 eV, while a triazine‐type host like DBFTrz has HOMO/LUMO around −6.5 to −6.7/−3.1 to −3.3 eV. A HOMO/LUMO offset of about 0.4–0.8/0.5–0.9 eV exists between the two classes of host materials, but they do not produce any exciplexes. Therefore, CBP, mCBP, and DBFTrz can be used as materials for the electroplex host. A common mixed host and an exciplex host were used for comparison.

**Figure 1:**
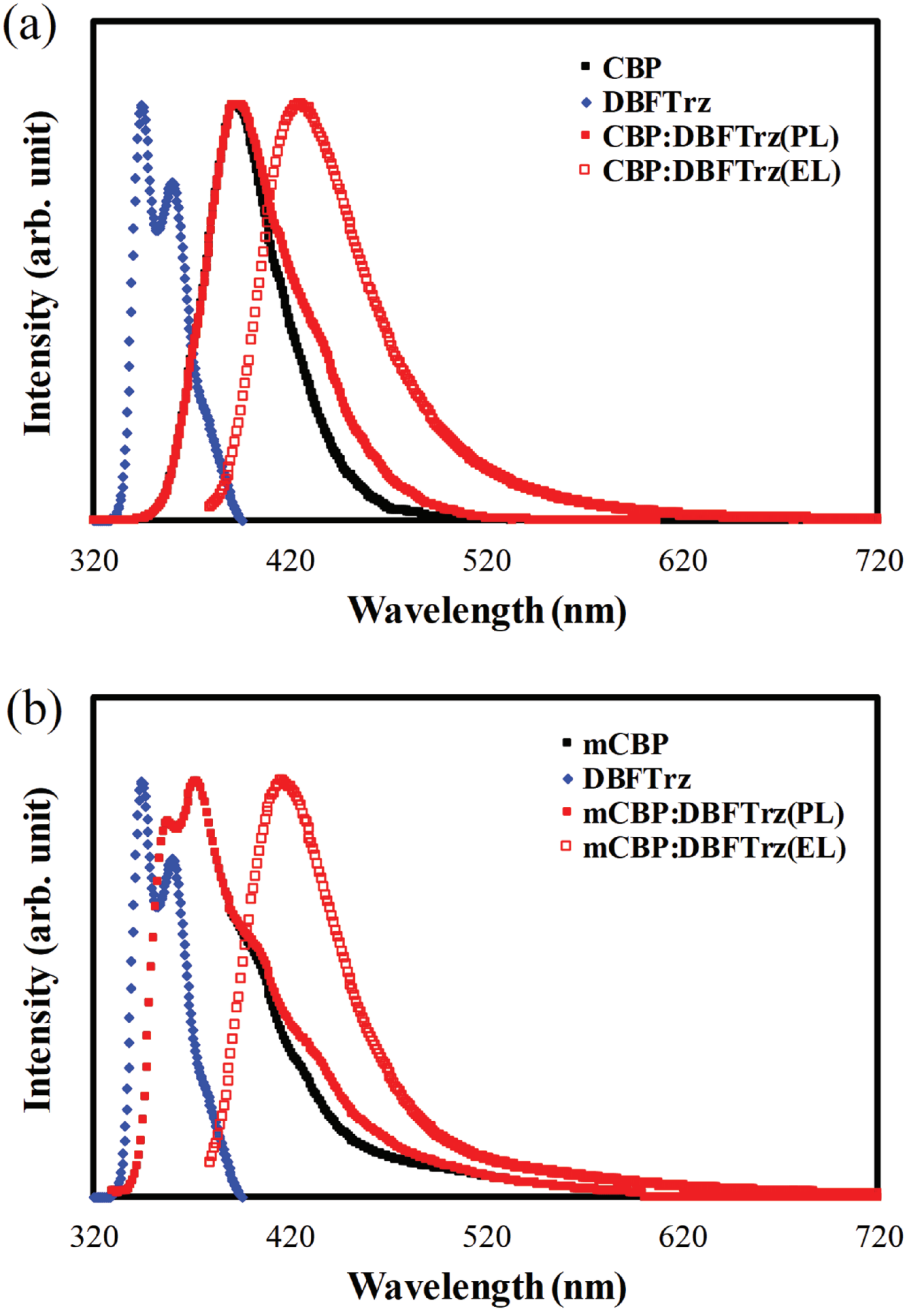
a) PL spectra of CBP, DBFTrz, and CBP:DBFTrz, and EL spectra of CBP:DBFTrz device. b) PL spectra of mCBP, DBFTrz, and mCBP:DBFTrz, and EL spectra of mCBP:DBFTrz device.

First, an electroplex host for red, yellow, green, and blue PhOLEDs was developed using CBP:DBFTrz and mCBP:DBFTrz. The CBP:DBFTrz was devised for red, yellow, and green devices, while the mixed host of mCBP:DBFTrz was developed for blue PhOLEDs. The mCBP and DBFTrz have triplet energies higher than 2.8 eV and are good electroplex host candidates for blue triplet emitters because the risk of reverse energy transfer from an Ir emitter to the host is low. The electroplex formation in the CBP:DBFTrz and mCBP:DBFTrz mixed hosts was studied by comparing the photoluminescence (PL) and electroluminescence (EL) emission of the CBP:DBFTrz and mCBP:DBFTrz mixed hosts. **Figure** **1**a,b shows PL and EL spectra of CBP:DBFTrz and mCBP:DBFTrz along with PL spectra of each host comprising the mixed host. The PL spectrum of CBP:DBFTrz was quite similar to that of the CBP solid film, but the EL spectrum was a featureless spectrum covering a wide range of wavelengths. This indicates that the EL emission is produced by an intermolecular complex, as exemplified in other exciplexes.^11^, ^12^ Featureless and broad emission was observed only in the EL spectrum under an electric field, confirming the electroplex formation between CBP and DBFTrz. Similarly, mCBP:DBFTrz also showed broad EL emission spectrum, which is shifted to long wavelength compared to the emission of mCBP and DBFTrz. The absence of the broad emission in the PL spectrum of mCBP:DBFTrz supports the electroplex formation in the mCBP:DBFTrz host. Composition‐dependent EL spectra of CBP:DBFTrz and mCBP:DBFTrz are shown in the Supporting Information and support the electroplex formation (Figure S1, Supporting Information).

**Figure 2:**
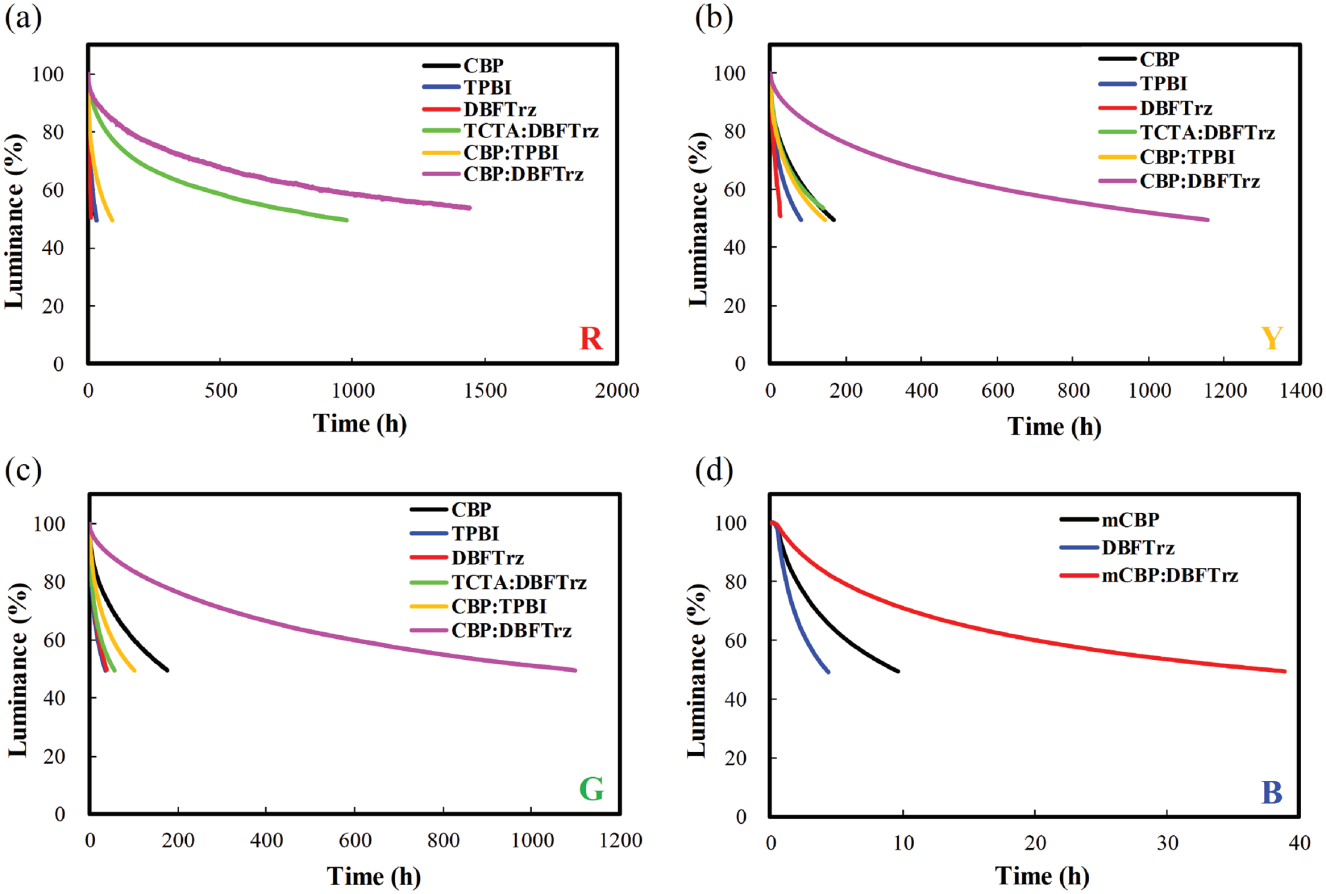
Lifetime curve of a) red, b) yellow, and c) green devices using the CBP, TPBI, DBFTrz, CBP:TPBI, TCTA:DBFTrz, and CBP:DBFTrz devices at 1000 cd m^−2^. Lifetime curve of d) blue devices using the mCBP, DBFTrz, mCBP:DBFTrz devices at 500 cd m^−2^.

The lifetimes of red, yellow, and green PhOLEDs were studied using CBP, DBFTrz, and CBP:DBFTrz hosts, while the lifetime of blue PhOLEDs was investigated using mCBP, DBFTrz, and mCBP:DBFTrz. The lifetime data are described in **Figure** **2**. Red emitter was bis[2‐(3,5‐dimethylphenyl)‐4‐methyl‐quinoline](acetylacetonate)iridium(III) (Ir(mphmq)_2_tmd), yellow emitter was bis[4‐phenylthieno[3, 2‐c]pyridine](acetylacetonate)iridium(III) (PO‐01), green emitter was bis[2‐(2‐pyridinyl‐*N*)phenyl‐C](acetylacetonato)iridium(III) (Ir(ppy)_2_acac), and blue emitter was fac‐tris(3‐(1‐(2,6‐diisopropylphenyl)‐1H‐imidazol‐2‐yl)benzonitrile) iridium (Ir(CNpi)_3_). The lifetime of CBP:1,3,5‐tris(*N*‐phenylbenzimidazole‐2‐yl)benzene (TPBI) (conventional exciplex free mixed host) and tris(4‐carbazoyl‐9‐ylphenyl)amine (TCTA):DBFTrz (exciplex host) devices was also added for comparison. Energy level diagram of the devices and chemical structures of the materials are in Figure S2 in the Supporting Information. The lifetime of the CBP:DBFTrz mixed host device was much longer than that of a CBP and a DBFTrz single host device in all red, yellow, and green PhOLEDs. A more than fourfold increase of the lifetime was achieved using the electroplex type CBP:DBFTrz mixed host compared to the single host. Moreover, the lifetime of the CBP:DBFTrz electroplex host device was much longer than that of the common CBP:TPBI and TCTA:DBFTrz exciplex mixed host devices. The QE of PhOLEDs was also improved by the CBP:DBFTrz electroplex host (**Table** **1**; Figure S3, Supporting Information), demonstrating superiority of the electroplex host for high efficiency and long lifetime PhOLEDs. The same result was obtained in the mCBP:DBFTrz electrolex‐based blue PhOLEDs. The lifetime and device data confirmed that the mCBP:DBFTrz electroplex host effectively extends the lifetime of blue PhOLEDs by four times while simultaneously improving the QE of the device.

**Table 1:** The OLED devices performances using the single and mixed hosts

Dopant	Host	Voltage [V]a)	CIEa)	EQE [%]b)	PE [lm W^−1^]b)	CE [cd A^−1^]b)	LT_50_ a)
Ir(mphmq)_2_tmd	TCTA	10.2	0.65, 0.35	13.1/17.7	6.1/21.5	19.7/26.6	0.5
	CBP	10.2	0.65, 0.35	10.1/15.3	4.6/15.7	14.9/21.0	32.3
	TPBI	7.0	0.65, 0.35	16.2/19.2	10.6/24.0	23.6/27.3	33.2
	DBFTrz	7.6	0.65, 0.35	14.2/16.0	8.4/17.4	20.4/22.7	12.0
	TCTA:DBFTrz	7.7	0.65, 0.35	20.1/21.2	12.1/22.2	29.5/31.0	978.2
	CBP:TPBI	7.2	0.65, 0.35	15.8/19.1	10.1/24.2	23.2/27.3	92.0
	CBP:DBFTrz	7.8	0.65, 0.35	18.7/21.2	11.1/23.4	27.6/30.6	1443.6
PO‐01	TCTA	8.8	0.49, 0.50	10.9/13.7	12.2/22.5	34.0/42.9	1.9
	CBP	9.6	0.51, 0.49	18.3/21.0	17.7/38.1	53.9/65.1	168.6
	TPBI	6.7	0.51, 0.49	17.2/18.0	23.7/37.4	50.3/52.7	82.2
	DBFTrz	7.7	0.51, 0.49	18.0/19.8	22.4/41.1	54.5/59.7	27.8
	TCTA:DBFTrz	7.0	0.50, 0.49	18.5/18.6	25.2/36.6	56.0/56.5	141.1
	CBP:TPBI	6.9	0.51, 0.48	16.5/19.0	21.7/43.9	47.8/54.9	145.4
	CBP:DBFTrz	7.9	0.51, 0.49	19.7/22.1	23.3/45.2	58.4/66.1	1155.7
Ir(ppy)_2_acac	TCTA	8.8	0.30, 0.62	8.6/11.4	10.9/22.6	30.4/40.6	0.6
	CBP	9.8	0.34, 0.61	11.8/16.2	13.7/34.3	42.8/58.5	175.8
	TPBI	7.5	0.35, 0.61	12.2/14.2	18.5/39.3	44.1/51.4	36.4
	DBFTrz	7.9	0.35, 0.61	13.8/15.5	20.3/38.8	50.9/57.3	39.2
	TCTA:DBFTrz	6.9	0.33, 0.62	14.9/15.0	24.7/35.9	54.0/54.7	56.9
	CBP:TPBI	7.1	0.35, 0.61	12.5/14.4	19.9/40.7	44.9/51.8	101.7
	CBP:DBFTrz	7.9	0.35, 0.61	16.1/18.1	23.3/47.6	58.8/66.2	1098.6
Ir(CNpi)_3_	mCBP	5.9	0.15, 0.24	10.1/12.8	9.0/14.5	16.9/21.5	9.7
	DBFTrz	5.8	0.16, 0.30	12.5/12.7	13.3/18.3	24.4/24.8	4.4
	mCBP:DBFTrz	5.6	0.16, 0.29	17.8/18.0	19.5/23.2	34.6/34.8	38.9

^a)^ Red, yellow, and green devices; at 3000 cd m^−2^/blue devices; at 500 cd m^−2^

^b)^ Specific luminance^a)^/maximum; EQE: external quantum efficiency, PE: power efficiency, CE: current efficiency.

**Figure 3:**
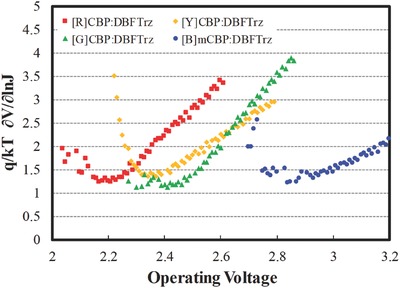
Ideality factor–voltage curves of the red, yellow, green, and blue devices using the electroplex mixed hosts (CBP:DBFTrz and mCBP:DBFTrz).

**Figure 4:**
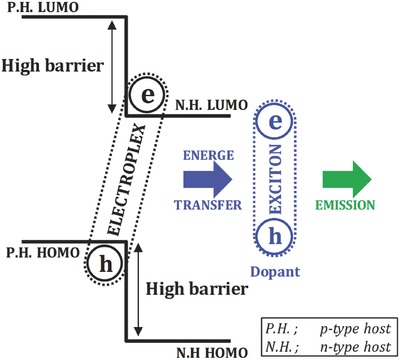
Schematic diagram showing the emission mechanism of electroplex type device.

The extended lifetime provided by the electroplex type mixed host was further investigated by analyzing the light emission mechanism. Previous investigators have demonstrated that charge trapping in the CBP:TPBI device is the major emission mechanism by fitting the ideal diode equation.^13^ The same method was used to analyze the emission mechanism of the electroplex host. **Figure** **3** shows the ideality factor fitting data of the electroplex devices. The ideality factor calculated from the current density–voltage–luminance plot of the CBP:DBFTrz and mCBP:DBFTrz electroplex host devices was close to 1 irrespective of the triplet emitters, which suggests that energy transfer rather than charge trapping dominates the light emission process of the electroplex host. Based on this result, the light emission process of the CBP:DBFTrz electroplex host device can be mainly described as hole injection and transport through the CBP or mCBP host, electron injection and transport through the DBFTrz host, electroplex formation between CBP and DBFTrz, and energy transfer from the electroplex to triplet emitters. This process is schematically pictured in **Figure** **4**. In the energy transfer mechanism, the number of positive and negative polarons in the host and triplet emitter is reduced by recombination of polarons and less charge trapping by the dopant. Because the main emission process of the CBP:DBFTrz device is energy transfer, the CBP:DBFTrz electroplex device is less susceptible to polaron‐related degradation processes like TPA. This may result in the longer observed PhOLED lifetimes.

The energy transfer process of the electroplex host was further confirmed by delayed EL measurements. A detailed description of the delayed EL measurement setup and signal detection protocol is reported elsewhere.^14^ In this technique, a device is driven using a square pulse driving scheme with a pulse width of 0.5 ms (the pulse is long enough for prompt EL to reach its steady‐state intensity). An optical shutter opens to collect a delayed EL around 0.1–0.3 ms after the end of the forward bias pulse, which is significantly longer than the lifetime of the Ir(ppy)_2_acac triplet state lifetime (<1 µs). This ensures the absence of any contributions from the prompt EL in the collected signal. As such, any collected signal will arise from the radiative decay of excitons that are formed after the end of the forward bias pulse. In general, the delayed EL can arise from one or several of the following processes: (i) recombination of residual (trapped) charges in the EML that become de‐trapped, and hence become capable of recombination and the emission of light, after the end of the forward bias pulse; (ii) slow migration of host excitons to the proximity of guest molecules and subsequent host to guest energy transfer; and (iii) triplet–triplet annihilation due to the fusion of host triplet excitons. Processes (ii) and (iii) are generally significant in devices where electroluminescence occurs via e–h on the host, followed by host‐to‐guest energy transfer. In contrast, process (i) is dominant in devices where electroluminescence occurs directly via e–h recombination on the dopant, which can leave more charges trapped on dopant sites.^15^

**Figure 5:**
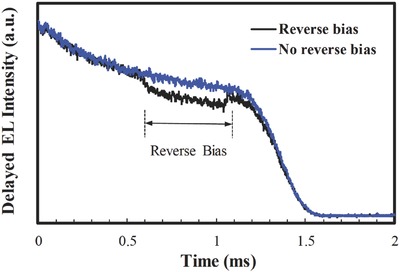
Time‐resolved delayed EL intensity of CBP:DBFTrz device without (blue) and with (black) the reverse bias voltage during the measurement. The applied reverse bias voltage is −5 V with a pulse time of 0.5 ms.

To test if process (i) is significant, a 0.5 ms reverse bias pulse (of −5 V magnitude) was applied during the delayed EL signal collection, and its effect on the delayed EL intensity was monitored. In our previous work, we showed that, in devices where process (i) is dominant, the application of the reverse bias leads to the appearance of a spike in delayed EL. This spike is an indication of increased e–h recombination of the de‐trapped charges under the influence of the reverse bias.^16^ As can be seen in **Figure** **5**, the delayed EL signal does not show a spike at the beginning of the reverse bias, which suggests that direct e–h recombination on the dopant plays an insignificant role in the device emission mechanism. Rather, the delayed EL intensity decreases by only a small amount at the beginning of the reverse bias and rebounds at the end of the reverse bias, a behavior that can be mainly attributed to electric‐field‐induced quenching of host excitons. This temporary reduction in the delayed EL intensity during the reverse bias pulse and the recovery at the end of the bias is an indication of TTA in the host triplets. Note that dopant–dopant TTA is unlikely to occur in the millisecond timeframe of our measurements. Therefore, any TTA will be mainly due to the fusion of long‐lived host triplets, indicating the presence of a high concentration of host excitons. The results confirm that e–h recombination occurs on the electroplex‐forming host and is followed by energy transfer to the dopant; this process is similar to that observed in other exciplex‐forming hosts.^17^

**Figure 6:**
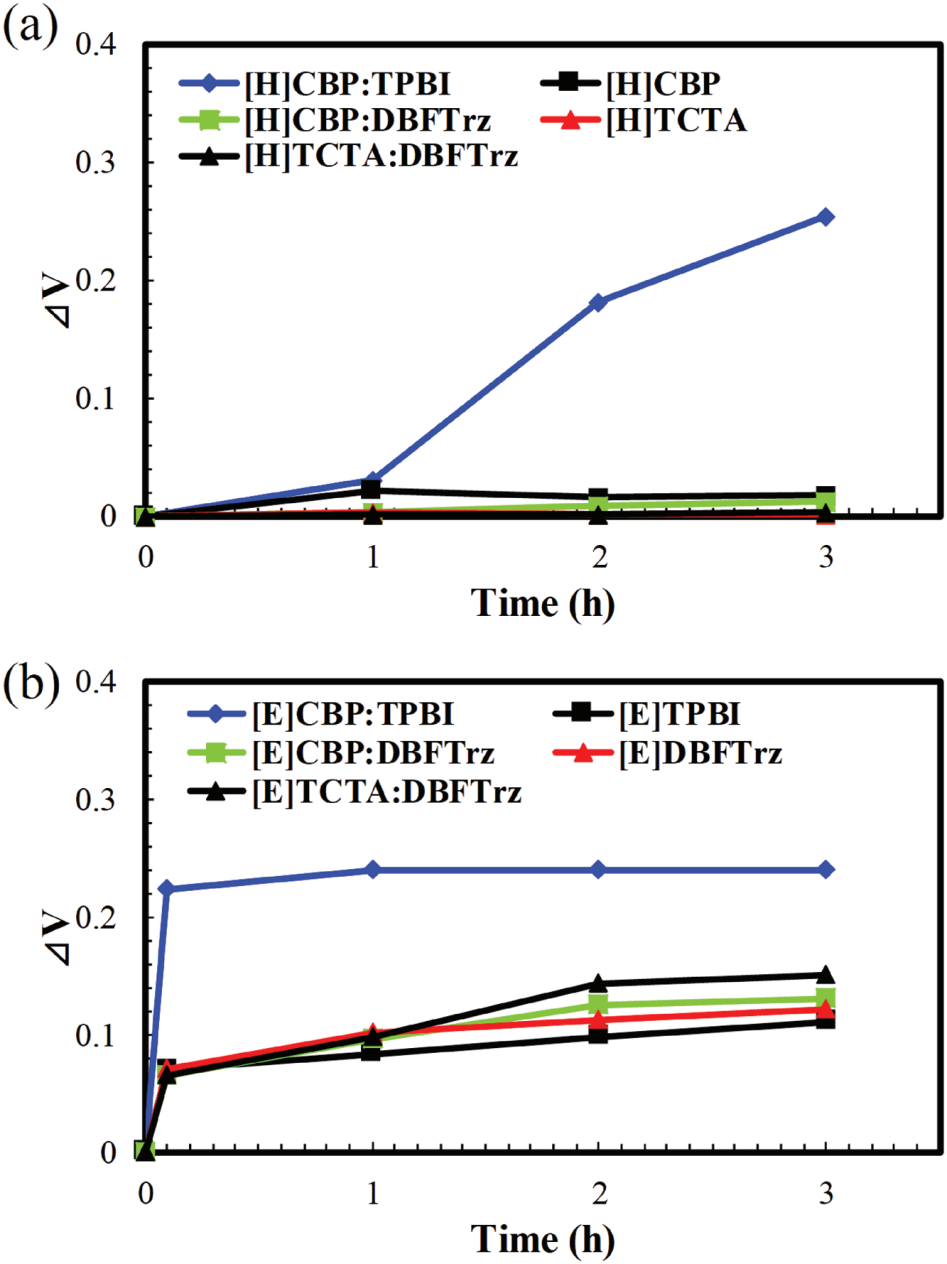
a) Voltage change of hole‐only devices of CBP, TCTA, TCTA:DBFTrz, CBP:TPBI, and CBP:DBFTrz devices according to operation time. b) Voltage change of electron‐only devices of TPBI, DBFTrz, TCTA:DBFTrz, CBP:TPBI, and CBP:DBFTrz devices according to operation time. Current density of the device test was 10 mA cm^−2^.

The single carrier stability of the electroplex host was measured to further study the lifetime extension provided by the electroplex host. Hole‐only and electron‐only devices of the CBP:TPBI and CBP:DBFTrz mixed hosts were aged under a current to assess the stability under positive and negative polarons. Aging test data of the single carrier devices are shown in **Figure** **6**. The hole carrier stability data of CBP:DBFTrz, CBP:TPBI, and TCTA:DBFTrz suggest that the CBP:DBFTrz electroplex host is comparable to TCTA:DBFTrz exciplex host and much more stable than the CBP:TPBI host because the voltage increase during driving for the hole‐only devices implies instability of the host under hole stress. The electron aging test of the electron‐only devices showed that the CBP:DBFTrz is superior to CBP:TPBI and TCTA:DBFTrz. The better single carrier stability of CBP:DBFTrz electroplex host than CBP:TPBI can be interpreted by the HOMO/LUMO offset between two host materials in the mixed host system. In the case of the CBP:DBFTrz electroplex host, the large HOMO/LUMO offset of 0.8/0.5 eV isolates holes in the CBP host and electrons in the DBFTrz host. Therefore, the electroplex host can be stable in the presence of both holes and electrons. However, the isolation of holes and electrons is incomplete in the CBP:TPBI host due to the small HOMO/LUMO offset of 0.2/0.2 eV, which destabilizes the CBP:TPBI host in the presence of both holes and electrons. This result clearly indicates that both hole and electron stability is ensured in the electroplex host. The hole stability of CBP:DBFTrz was similar to that of CBP, and the electron stability of CBP:DBFTrz was similar to that of DBFTrz. Comparing the CBP:DBFTrz electroplex host and TCTA:DBFTrz exciplex host, hole stability was similar, but the electron stability was better in the CBP:DBFTrz host than TCTA:DBFTrz host. As the HOMO/LUMO offset between two host materials in the mixed hosts was larger than 0.5 eV, the improved electron stability of the CBP:DBFTrz host can be explained by robustness of CBP under negative polarons relative to TCTA. In the electrical driving process, electrons can be injected into CBP or TCTA molecules in spite of large LUMO gap between two hosts. Most electrons may stay in DBFTrz, but some electrons may go to CBP or TCTA. The CBP or TCTA exposed to negative polarons can be degraded due to low bond dissociation energy (BDE) under negative polarons. Comparing the BDE of C (sp^2^)—N (sp^2^) bond of CBP and C (sp^2^)—N (sp^3^) of TCTA, the BDE of former C—N bond (36.9 kcal mol^−1^) is higher than that of the latter C—N bond (35.6 kcal mol^−1^) under negative polarons, which stabilizes CBP:DBFTrz relative to TCTA:DBFTrz.^18^ Both electroplex and exciplex hosts can suppress electron injection into the hole transport type hosts, but the hole transport type host in the electroplex host can be more robust than that in the exciplex host because the hole transport type host in the electrolex has relatively weak hole transport character. Therefore, it can be summarized that the extended lifetime of the electroplex host device is due to improved stability under hole and electron stress and an energy transfer emission mechanism.^4^

## 3 Conclusions

In conclusion, a CBP:DBFTrz electroplex host and an mCBP:DBFTrz electroplex host were developed as high efficiency and lifetime‐extending hosts for red, yellow, green, and blue PhOLEDs, respectively. These hosts improved the lifetime of red, yellow, green, and blue PhOLEDs by a factor of higher than four relative to control devices like single host, mixed host, and exciplex host devices. The light emission mechanism of the electroplex host was attributed to energy transfer rather than charge trapping by ideality factor analysis and delayed transient EL analysis. We found that the devices were stable in the presence of holes and electrons due to the large HOMO/LUMO offset and energy transfer emission mechanism. These were key factors that helped improve the lifetime of the electroplex host. The blue electroplex host was easy to prepare, unlike exciplex hosts, which are rather difficult to develop. Therefore, the electroplex type host can be useful for both high efficiency and long lifetime phosphorescent OLEDs.

## 4 Experimental Section

*Device Fabrication and Materials*: The red, yellow, and green devices employed indium tin oxide substrates (ITO) as an anode;*N*,*N*′‐diphenyl‐*N*,*N*′‐bis‐[4‐(phenyl‐*m*‐tolyl‐amino)‐phenyl]‐biphenyl‐4,4′‐diamine (DNTPD) as a hole injection layer; *N*,*N*,*N*′*N*′‐tetra[(1,1′‐biphenyl)‐4‐yl]‐(1,1′‐biphenyl)‐4,4′‐diamine (BPBPA) as a hole transport layer; 9,9‐dimethyl‐10‐(9‐phenyl‐9H‐carbazol‐3‐yl)‐9,10‐dihydroacridine(PCZAC) as an electron blocking layer; Ir(mphmq)_2_tmd, PO‐01, and Ir(ppy)_2_acac as red, yellow, and green phosphorescent dopants; CBP, mCBP, and TCTA as hole transport type hosts; DBFTrz and TPBI as electron transport type hosts; LiF as an electron injection layer; and Al as a cathode. The whole device structure was ITO (120 nm)/DNTPD (60 nm)/BPBPA (20 nm)/PCZAC (10 nm)/emitting layer (30 nm)/TPBI (35 nm)/LiF (1.5 nm)/Al (200 nm). The emitting layers of the devices were either single hosts of CBP, and DBFTrz or mixed hosts of CBP:TPBI, CBP:DBFTrz, and TCTA:DBFTrz doped with phosphorescent emitters. The ratio of the two hosts in the mixed hosts was 50:50, and the dopant concentration was 5 wt%.

The blue devices employed ITO as an anode, DNTPD as a hole injection layer, BPBPA as a hole transport layer, PCZAC as an electron blocking layer, Ir(CNpi)_3_ as a blue phosphorescent dopant, mCBP as a hole transport type host, DBFTrz as an electron transport type host, 2‐(4‐(9,10‐di(naphthalen‐2‐yl)anthracen‐2‐yl)phenyl)‐1‐phenyl‐1H‐benzo[d]imidazole (ZADN) as an electron transport layer, LiF as an electron injection layer, and Al as a cathode. The whole device structure was ITO (50 nm)/DNTPD (40 nm)/BPBPA (10 nm)/PCZAC (10 nm)/emitting layer (30 nm)/DBFTrz (5 nm)/ZADN (20 nm)/LiF (1.5 nm)/Al (200 nm). The emitting layers of the devices were either a single host of mCBP or DBFTrz or a mixed host of mCBP:DBFTrz doped with phosphorescent Ir(CNpi)_3_. The ratio of the two hosts in the mixed hosts was 50:50, and the dopant concentration was 5 wt%.

The hole‐only device structure was as follows: ITO (120 nm)/MoO_3_ (5 nm)/hole transport type host (20 nm)/mixed hosts (50 nm)/hole transport type host (20 nm)/MoO_3_ (5 nm)/Al (200 nm). The electron‐only device structure was as follows: ITO (120 nm)/electron transport type host (20 nm)/mixed hosts (50 nm)/electron transport type host (20 nm)/LiF(1.5 nm)/Al (200 nm). The electroplex device structure for transient EL was as follows: ITO (120 nm)/MoO_3_ (5 nm)/CBP:DBFTrz (80 nm)/LiF (1.5 nm)/Al (200 nm). The ratio of the two hosts in the mixed host was 50:50.

*Measurements*: The change in voltage of hole‐ and electron‐only devices was measured using a Keithley Series 2400 at an applied current density of 10 mA cm^−2^. PL measurements were carried out using 30‐nm‐thick films on a quartz substrate fabricated using a vacuum deposition process. The films were excited using a 320 nm light source generated in a fluorescence spectrometer (Perkin‐Elmer LS 55) employing a 40 W Xenon lamp.

## Conflict of Interest

The authors declare no conflict of interest.

## Supporting information

Supplementary
