# Supplementary material for: Electroplex as a New Concept of Universal Host for Improved Efficiency and Lifetime in Red, Yellow, Green, and Blue Phosphorescent Organic Light‐Emitting Diodes
Source: Adv Sci (Weinh). 2017 Dec 1;5(2):1700608. doi: 10.1002/advs.201700608 (PMC5827470; doi:10.1002/advs.201700608)
Supplement: Supplementary file 1 — Supplementary [file ADVS-5-1700608-s001.pdf]

Copyright WILEY-VCH Verlag GmbH & Co. KGaA, 69469 Weinheim, Germany, 2017.

ADVANCED SCIENCE Open Access

Supporting Information

for *Adv. Sci.,* DOI: 10.1002/advs.201700608

Electroplex as a New Concept of Universal Host for Improved Efficiency and Lifetime in Red, Yellow, Green, and Blue Phosphorescent Organic Light-Emitting Diodes

Wook Song, Jun Yeob Lee, Yong Joo Cho, Hyeonghwa Yu, Hany Aziz, and Kang Mun Lee*

Supporting information

Electroplex as a new concept of universal host for improved lifetime in red, yellow, green and blue phosphorescent organic light-emitting diodes

Wook Song[1], Jun Yeob Lee[1*], Yong Joo Cho[2], Hyeonghwa Yu[2], Hany Aziz[2], Kang Moon Lee[3]

[1]School of Chemical Engineering, Sungkyunkwan University
2066, Seobu-ro, Jangan-gu, Suwon, Gyeonggi, 16419, Korea

[2] Department of Electrical and Computer Engineering & Waterloo Institute for Nanotechnology, University of Waterloo, 200 University Avenue West, Waterloo, Ontario, Canada N2L 3G1

[3]Department of Chemistry, Kangwon University, 1, Kwongwondaehakgil, Chuncheon, Kangwon, 24341, Korea

E-mail: leej17@skku.edu

(a)

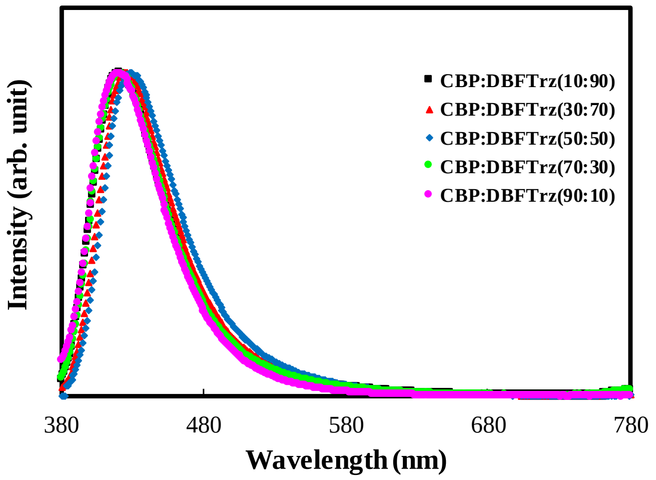

(b)

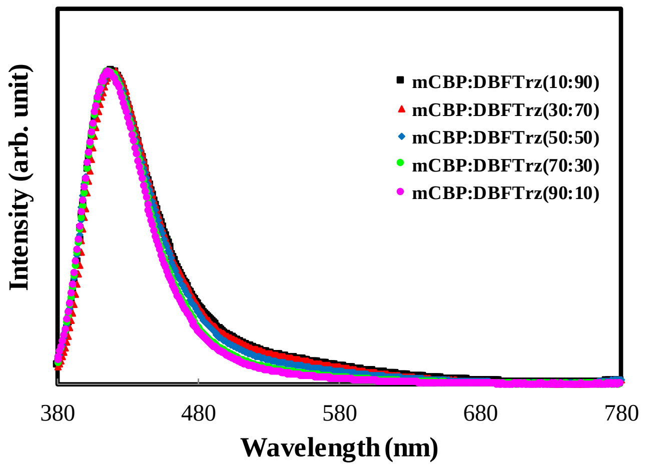

Figure S1. EL spectra of CBP:DBFTrz **(a)** and mCBP:DBFTrz **(b)** devices according to mixed host composition. The devices structure were ITO (120 nm)/MoO_3 (5 nm)/CBP:DBFTrz (80 nm) /LiF(1.5 nm)/Al (200 nm) and ITO (120 nm)/MoO_3 (5 nm)/mCBP:DBFTrz (80 nm) /LiF(1.5 nm)/Al (200 nm). The compositions of the mixed hosts (CBP:DBFTrz and mCBP:DBFTrz) were 10:90, 30:70, 50:50, 70:30, and 90:10.

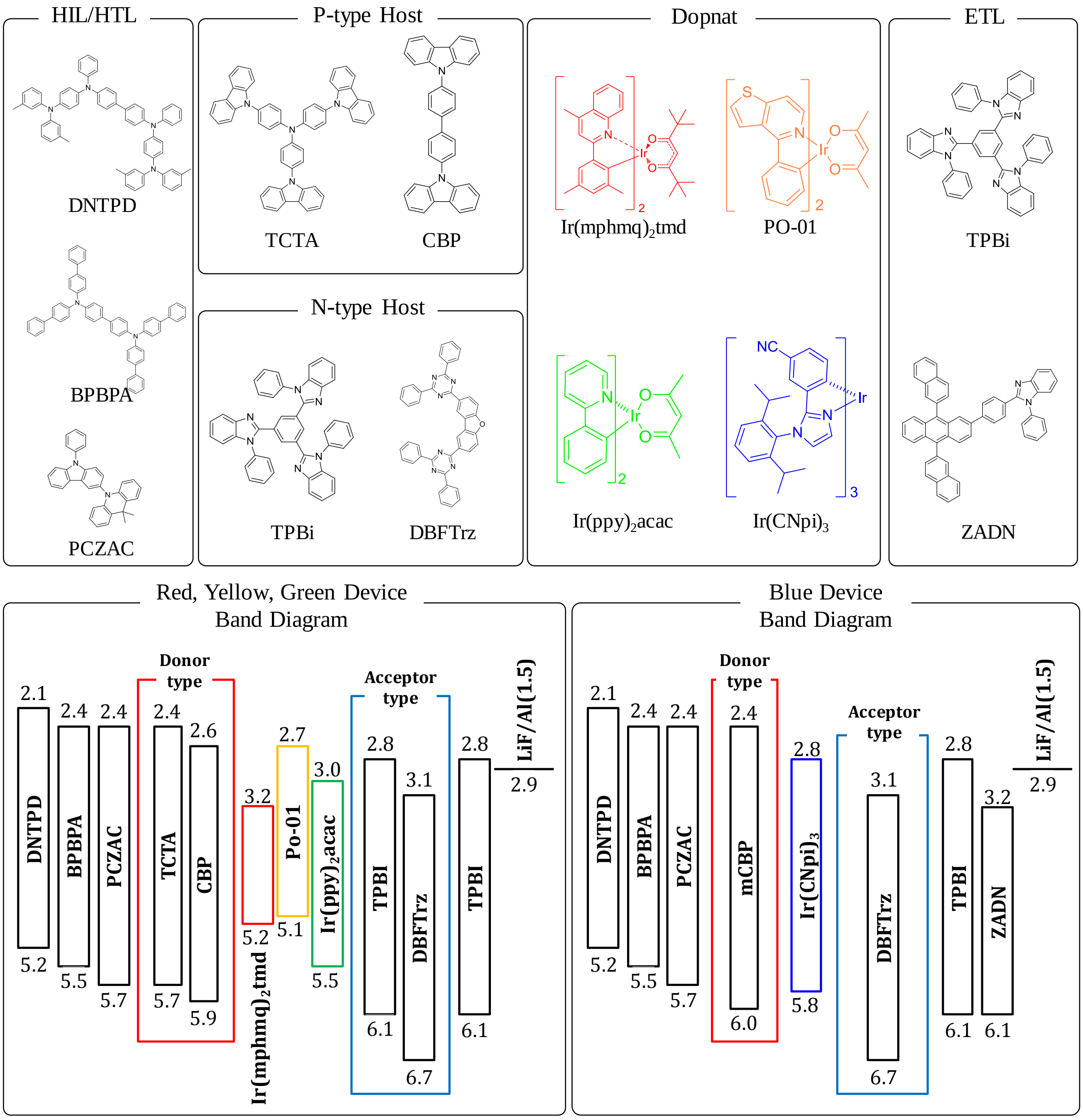

Figure S2. Chemical structures and band diagrams of materials in the phosphorescent devices.

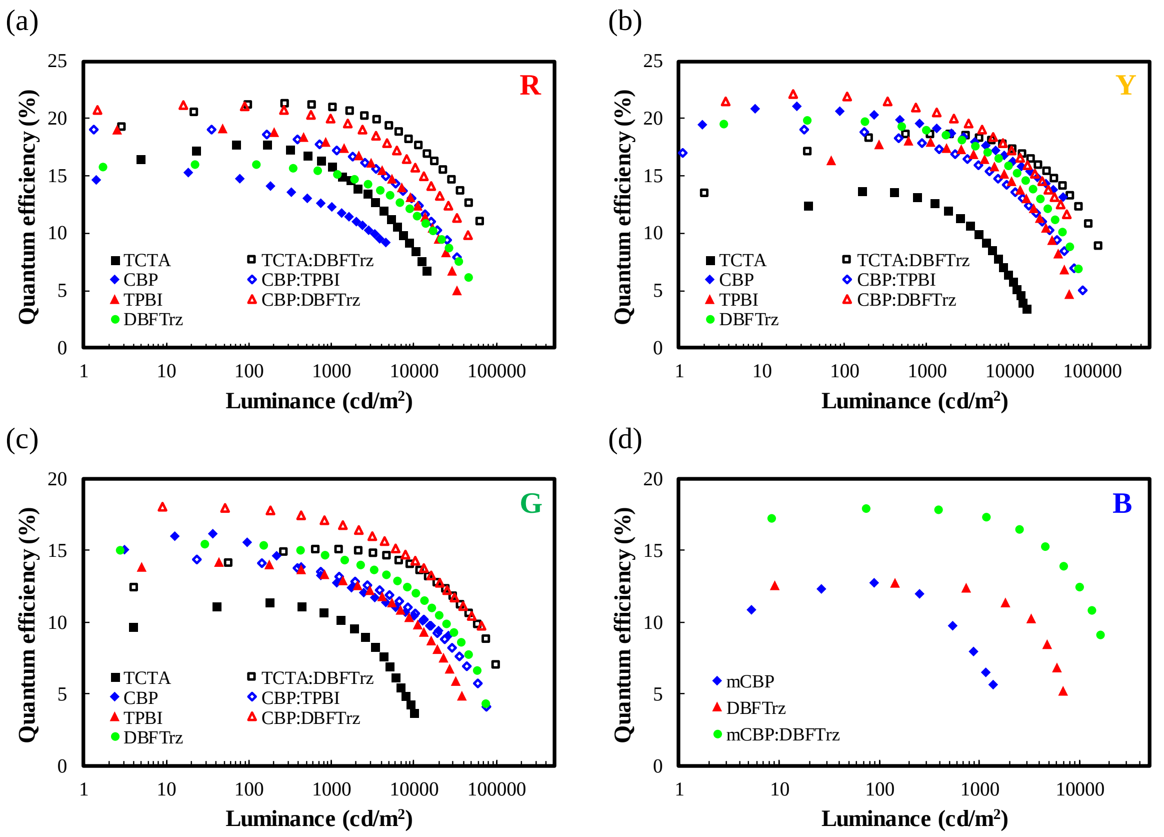

Figure S3. The external quantum efficiency of red **(a)**, yellow **(b)**, green **(c)** and blue **(d)** phosphorescent devices.
